# Supplementary figures and images for: Assessment of sealing efficacy, radiopacity, and surface topography of a bioinspired polymer for perforation repair
Source: PeerJ. 2024 Apr 29;12:e17237. doi: 10.7717/peerj.17237 (PMC11064857; doi:10.7717/peerj.17237)

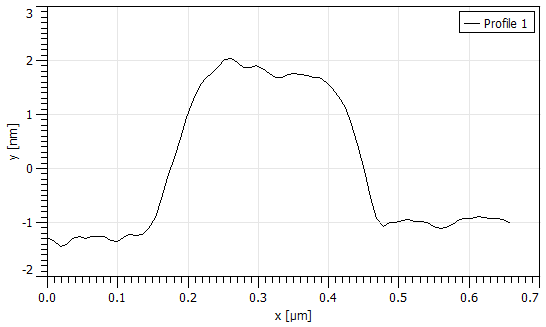

Supplement: Supplemental Information 4 [file peerj-12-17237-s004.zip › AFM data/1/1.bmp]

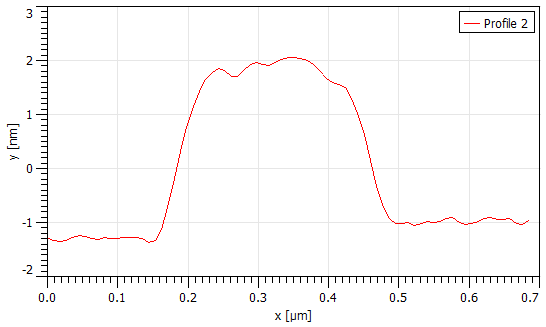

Supplement: Supplemental Information 4 [file peerj-12-17237-s004.zip › AFM data/1/2.bmp]

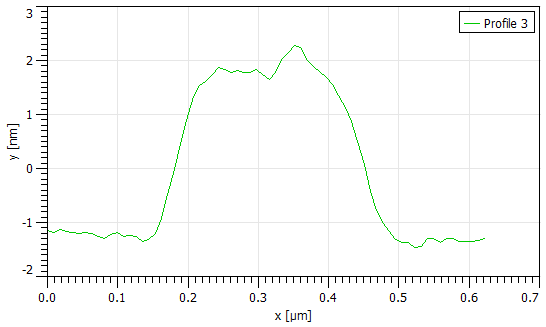

Supplement: Supplemental Information 4 [file peerj-12-17237-s004.zip › AFM data/1/3.bmp]

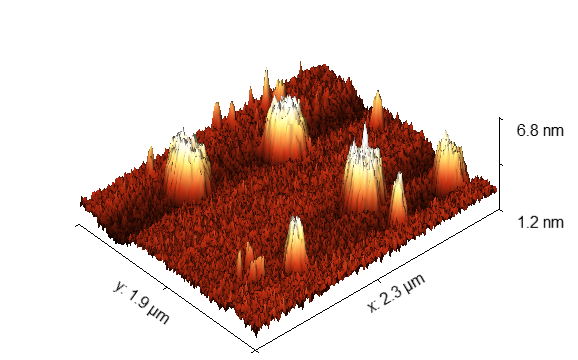

Supplement: Supplemental Information 4 [file peerj-12-17237-s004.zip › AFM data/1/3d.bmp]

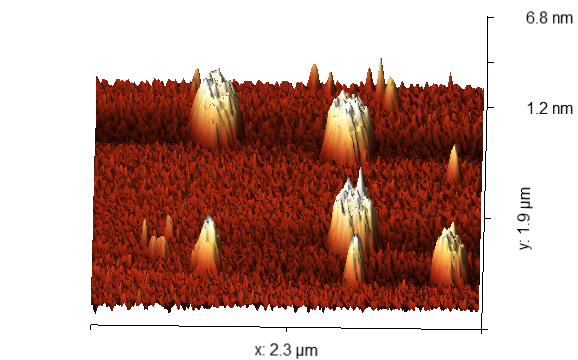

Supplement: Supplemental Information 4 [file peerj-12-17237-s004.zip › AFM data/1/3d1.bmp]

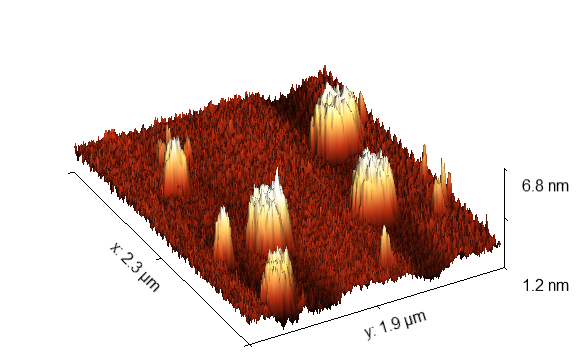

Supplement: Supplemental Information 4 [file peerj-12-17237-s004.zip › AFM data/1/3d2.bmp]

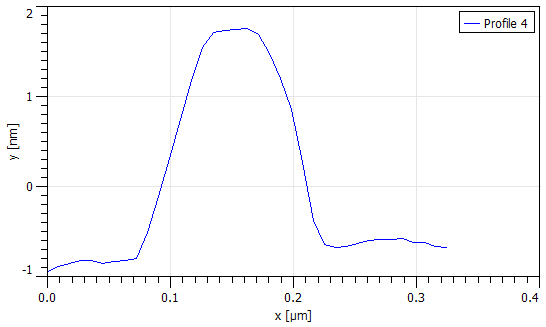

Supplement: Supplemental Information 4 [file peerj-12-17237-s004.zip › AFM data/1/4.bmp]

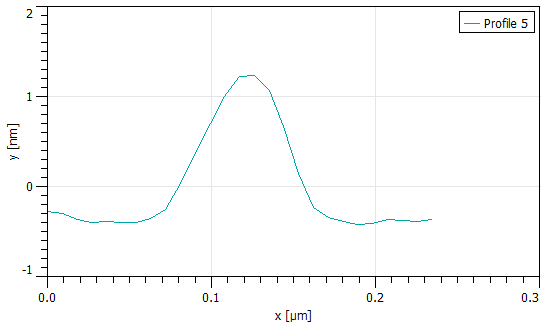

Supplement: Supplemental Information 4 [file peerj-12-17237-s004.zip › AFM data/1/5.bmp]

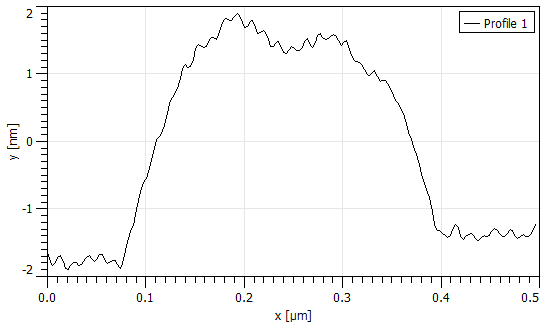

Supplement: Supplemental Information 4 [file peerj-12-17237-s004.zip › AFM data/2/1.bmp]

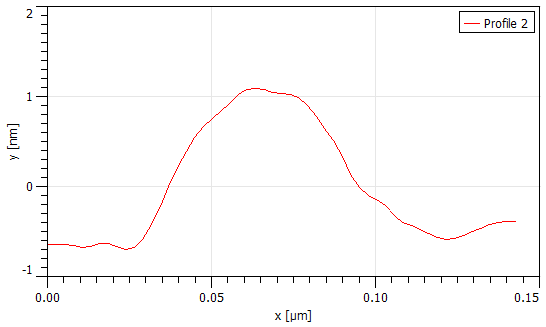

Supplement: Supplemental Information 4 [file peerj-12-17237-s004.zip › AFM data/2/2.bmp]

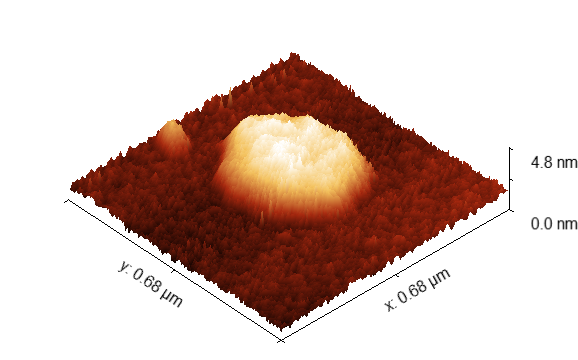

Supplement: Supplemental Information 4 [file peerj-12-17237-s004.zip › AFM data/2/3d.bmp]

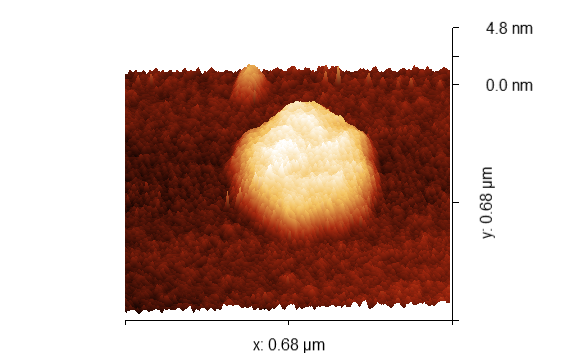

Supplement: Supplemental Information 4 [file peerj-12-17237-s004.zip › AFM data/2/3d1.bmp]

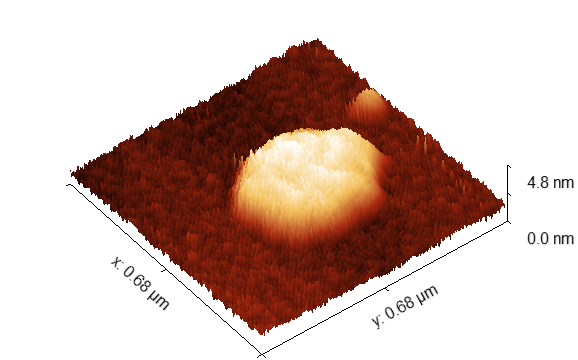

Supplement: Supplemental Information 4 [file peerj-12-17237-s004.zip › AFM data/2/3d2.bmp]

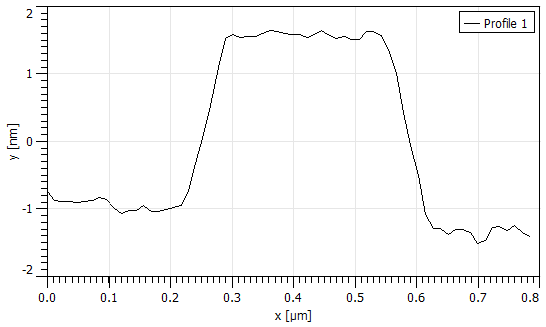

Supplement: Supplemental Information 4 [file peerj-12-17237-s004.zip › AFM data/3/1.bmp]

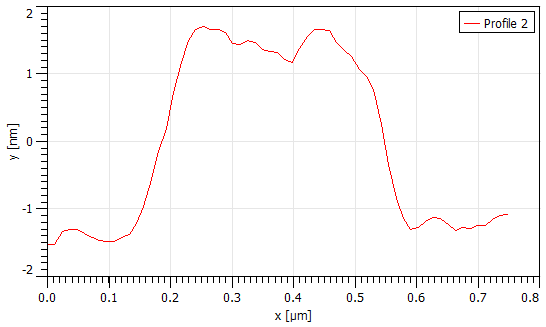

Supplement: Supplemental Information 4 [file peerj-12-17237-s004.zip › AFM data/3/2.bmp]

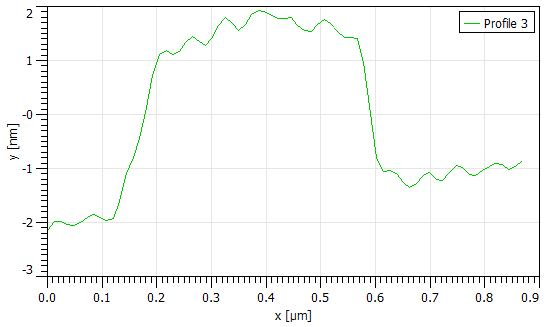

Supplement: Supplemental Information 4 [file peerj-12-17237-s004.zip › AFM data/3/3.bmp]

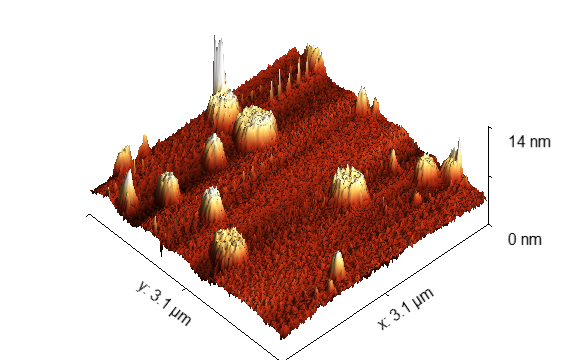

Supplement: Supplemental Information 4 [file peerj-12-17237-s004.zip › AFM data/3/3d.bmp]

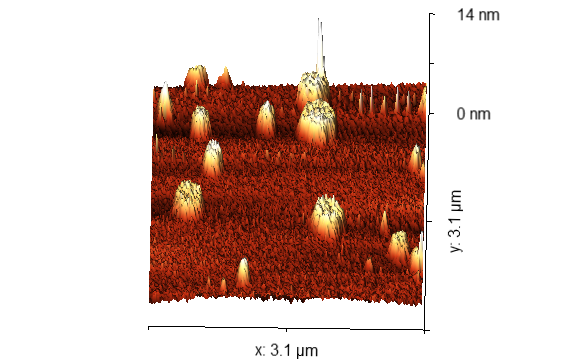

Supplement: Supplemental Information 4 [file peerj-12-17237-s004.zip › AFM data/3/3d1.bmp]

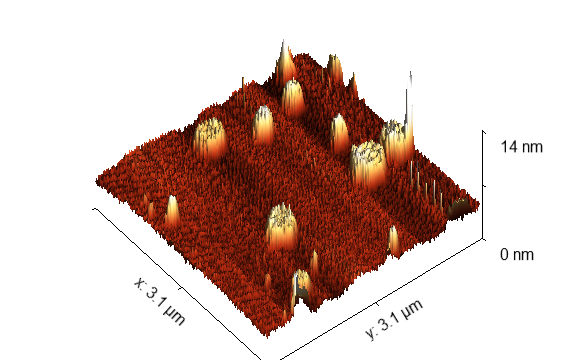

Supplement: Supplemental Information 4 [file peerj-12-17237-s004.zip › AFM data/3/3d2.bmp]

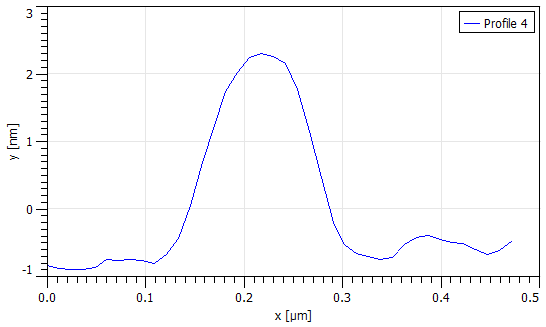

Supplement: Supplemental Information 4 [file peerj-12-17237-s004.zip › AFM data/3/4.bmp]

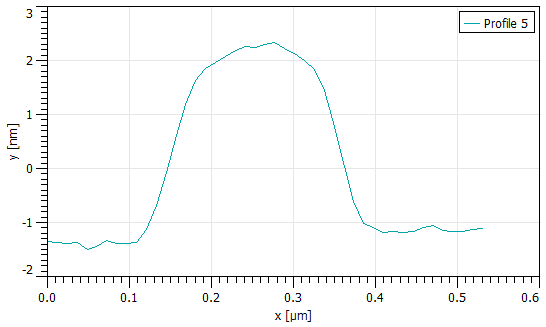

Supplement: Supplemental Information 4 [file peerj-12-17237-s004.zip › AFM data/3/5.bmp]
